# Surface Plasmon Resonance Immunosensor for Direct Detection of Antibodies against SARS-CoV-2 Nucleocapsid Protein

**DOI:** 10.3390/ijms25168574

**Published:** 2024-08-06

**Authors:** Viktorija Lisyte, Asta Kausaite-Minkstimiene, Benediktas Brasiunas, Anton Popov, Almira Ramanaviciene

**Affiliations:** NanoTechnas–Center of Nanotechnology and Materials Science, Institute of Chemistry, Faculty of Chemistry and Geosciences, Vilnius University, Naugarduko Str. 24, LT-03225 Vilnius, Lithuania; viktorija.lisyte@chgf.stud.vu.lt (V.L.); asta.kausaite@chf.vu.lt (A.K.-M.); benediktas.brasiunas@chgf.vu.lt (B.B.)

**Keywords:** immunosensor, SARS-CoV-2 nucleocapsid protein, antibody, surface plasmon resonance

## Abstract

The strong immunogenicity of the SARS-CoV-2 nucleocapsid protein is widely recognized, and the detection of specific antibodies is critical for COVID-19 diagnostics in patients. This research proposed direct, label-free, and sensitive detection of antibodies against the SARS-CoV-2 nucleocapsid protein (anti-SCoV2-rN). Recombinant SARS-CoV-2 nucleocapsid protein (SCoV2-rN) was immobilized by carbodiimide chemistry on an SPR sensor chip coated with a self-assembled monolayer of 11-mercaptoundecanoic acid. When immobilized under optimal conditions, a SCoV2-rN surface mass concentration of 3.61 ± 0.52 ng/mm^2^ was achieved, maximizing the effectiveness of the immunosensor for the anti-SCoV2-rN determination. The calculated *K_D_* value of 6.49 × 10^−8^ ± 5.3 × 10^−9^ M confirmed the good affinity of the used monoclonal anti-SCoV2-rN antibodies. The linear range of the developed immunosensor was from 0.5 to 50 nM of anti-SCoV2-rN, where the limit of detection and the limit of quantification values were 0.057 and 0.19 nM, respectively. The immunosensor exhibited good reproducibility and specificity. In addition, the developed immunosensor is suitable for multiple anti-SCoV2-rN antibody detections.

## 1. Introduction

An extensive worldwide outbreak of the virus known as severe acute respiratory syndrome coronavirus 2 (SARS-CoV-2) has left a profound imprint on both global public health and the international economy [1]. Since the SARS-CoV-2 virus was first identified in Wuhan in December 2019, it has caused more than 775 million officially reported COVID-19 cases as of June 2024. Unfortunately, this global health crisis has also resulted in a staggering number of confirmed death—more than seven million [2]. SARS-CoV-2 was found to consist of four main structural proteins. Three of them are integrated into the virus lipid membrane: the spike (S) protein, envelope (E) protein, and membrane (M) protein. In addition, an essential nucleocapsid (N) protein (RNA-binding protein) is present in the SARS-CoV-2 structure [3]. After infection, the N protein enters the host cell together with viral RNA, playing a key role in enhancing and regulating RNA replication, as well as controlling the assembly and subsequent exit of viral particles. Notably, the N protein is highly immunogenic and capable of abundant expression throughout the infectious process [4]. The N protein serves as a versatile RNA-binding protein essential for viral RNA transcription and replication processes. It assumes a variety of crucial functions, including the formation of helical ribonucleoproteins while packaging the viral RNA genome, governing viral RNA synthesis during replication and transcription, and influencing the metabolic activities of infected cells [5].

Antibody production is an important part of the immune response [6]. Quantification of antibodies against SARS-CoV-2 virus proteins has become an crucial part of the pandemic strategy [7]. During infection, antibodies against SARS-CoV-2 virus proteins are produced in the human body, with the greatest number of specific antibodies directed against the S and N proteins [8]. The concentration of specific antibodies against the N protein is particularly high in the serum of patients after prolonged hospitalization [9]. Specific antibodies are determined in the blood for a long time, although, of course, their titer decreases. Given the fact that the N protein, like other SARS-CoV-2 virus structural proteins, is detectable in most people up to 12 weeks after infection [10], antibody detection becomes the only way to determine whether a person has been infected with SARS-CoV-2. Although protein S has been used as a target for the most developed vaccines, the detection of specific antibodies against the N protein allows isolation of a case of the disease even among vaccinated individuals. In addition, a correlation between the level of antibodies against the N protein and the virus-neutralizing potential of serum was shown, but it was judged to be moderate [11]. The N protein was offered as a target for antiviral drugs; moreover, it can be useful for vaccine development [12,13]. Therefore, antibodies against the N protein may play a role as effective markers of exposure [11]. Thus, the detection of these antibodies is an important analytical challenge.

As an immune response to the SARS-CoV-2 virus, various antibodies against the N protein are produced in the body. Among them, specific immunoglobulin G (IgG) antibodies can be detected as early as 8 days after infection, and their high concentration can persist for a long time, but begins to decrease with time [14]. The concentration of antibodies and their decline over time varies from person to person [15]. Various methods, such as serological tests [16], enzyme-linked immunosorbent assay (ELISA) [17,18], dissociation-enhanced lanthanide fluorescence immunoassay [19], electrochemical immunoassay [14,20,21], lateral flow immunoassay [22], electrochemiluminescence immunoassay [23], fluorescent immunochromatography assay [24], surface plasmon resonance (SRP) spectroscopy [25,26,27], etc., have been recently used to detect antibodies against the N protein.

SPR spectroscopy is a sensitive technique that provides real-time label-free detection in relatively small volumes [28] from complex samples, without using sample pretreatment [29]. The sensitivity of SPR spectroscopy is particularly high when using a direct detection format in the case of high molecular weight biomolecules, such as antibodies [30]. The response time of the SPR immunosensors of less than 15 min makes this technique preferable to methods such as ELISA [31]. The high price of the instruments, which used to be one of the disadvantages of the SPR methodology, has decreased significantly recently with the appearance of small portable devices with sufficient sensitivity to detect analytes at low concentrations [29]. The use of SPR immunosensors based on optical fibers is another promising direction for sensitive low-cost detection [32].

**Figure 1:**
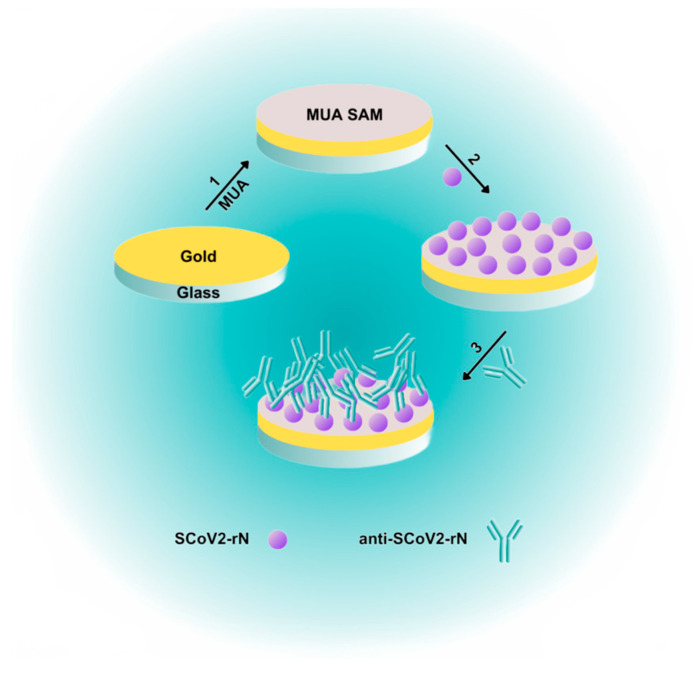
Simplified schematic illustration of SPR immunosensor fabrication and direct anti-SCoV2-rN detection. (1) Formation of MUA SAM on the surface of the SPR sensor chip. (2) Covalent immobilization of SCoV2-rN using carbodiimide conjugation chemistry. (3) Interaction of immobilized SCoV2-rN with specific antibodies—anti-SCoV2-rN.

This work describes the development and study of a SPR immunosensor for the real-time determination of specific antibodies against the SARS-CoV-2 N protein (Figure 1). The focus has been on developing a simple and sensitive immunosensor, ensuring relatively rapid detection of antibodies against the N protein. The recombinant SARS-CoV-2 N protein (SCoV2-rN) was immobilized on the surface of the SPR sensor chip coated with a self-assembled monolayer (SAM) of 11-mercaptoundecanoic acid (MUA) and used for the direct and sensitive detection of mouse monoclonal antibodies against SCoV2-rN (anti-SCoV2-rN). The analytical performance of the fabricated immunosensor was assessed.

## 2. Results

**Figure 2:**
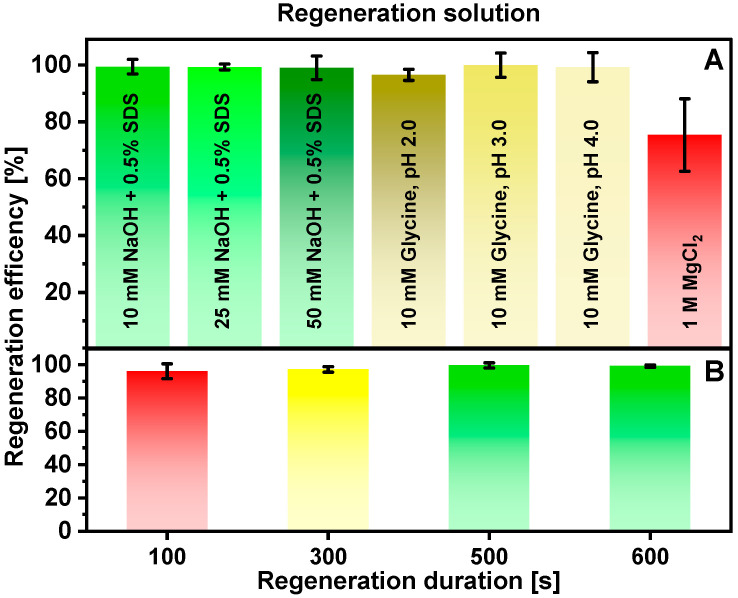
Dependence of Au/SCoV2-rN/anti-SCoV2-rN complex dissociation and surface regeneration efficiency on the (**A**) nature of the regeneration solution and (**B**) regeneration duration using 10 mM NaOH and 0.5% SDS solution. Conditions: 500 nM SCoV2-rN; 10 nM anti-SCoV2-rN. Error bars represent the standard deviation of three measurements (n = 3).

SPR spectroscopy is a powerful technique that provides sensitive, label-free analyte detection. Optimization of the regeneration procedure is necessary to ensure the reliability and reproducibility of the immunosensor. In addition, the reusability of the pre-modified SPR sensor chip results in lower measurement costs. Disruption of the antibody–antigen immune complex formed on the SPR sensor chip can be achieved during the surface regeneration process by overcoming the forces of attraction. For this purpose, detergents, strong electrolytes, and acidic or basic solutions are used, making sure that there are no irreversible changes in the structure of the immobilized molecules [33,34]. The efficiency of seven commonly used regeneration solutions was tested (Figure 2A).

After immune complex formation and the subsequent dissociation phase, regeneration was performed for 500 s. The results showed that the lowest regeneration efficiency (75.3 ± 12.8%) with low repeatability was observed when 1 M MgCl_2_ solution was used. It can be assumed that solutions of strong electrolytes with high ionic strength are not preferable for the regeneration of the antigen–antibody pair used. Solutions consisting of 10 mM NaOH and 0.5% SDS and 25 mM NaOH + 0.5% SDS showed similar regeneration efficiencies of 99.4 ± 2.5% and 99.3 ± 1.0%, respectively. The use of 10 mM glycine, pH 3.0, and 10 mM glycine, pH 4.0, solutions achieved comparable efficiencies, but it was noted that the registered analytical signals gradually decreased with the repeated use of these acidic solutions. Although the study showed no statistical difference in the results depending on the regeneration solution used, except for the use of MgCl_2_ solution, the smallest standard deviation was calculated for experiments using the 10 mM NaOH and 0.5% SDS solution. For further experiments, a solution of 10 mM NaOH and 0.5% SDS was chosen to reduce possible inactivation of immobilized SCoV2-rN protein.

The efficiency also depends on the duration of regeneration. The optimal duration of regeneration was determined by exposing the formed immune complex to 10 mM NaOH and 0.5% SDS solution for various periods of time (Figure 2B). After 100 s of regeneration, an efficiency of 96.0 ± 4.4% was achieved. An increase in regeneration efficiency to 99.6 ± 1.5% was monitored, and then regeneration was prolonged to 500 s. Longer regeneration had no positive effect on regeneration efficiency. Although the study showed no statistically significant difference in regeneration efficiency, considering the average efficiency values, and trying to avoid a possible irreversible reduction in the immunosensor signal, a 500 s regeneration duration was chosen as optimal.

**Figure 3:**
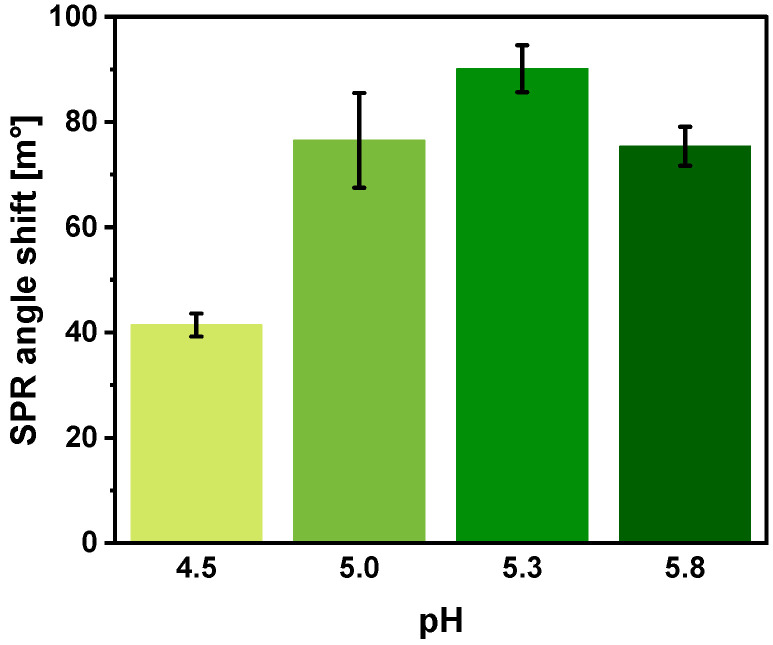
Effect of the pH value of acetate buffer used for SCoV2-rN dilution on the magnitude of the response of SCoV2-rN and anti-SCoV2-rN immune complex formation. Conditions: 500 nM SCoV2-rN; immobilization duration—1200 s; 15 nM anti-SCoV2-rN; 50 mM acetate buffers with different pH. Error bars represent the standard deviation of three measurements (n = 3).

SCoV2-rN protein immobilization was performed using carbodiimide conjugation chemistry by activating carboxyl groups present on the Au/MUA surface with a mixture of EDC/NHS. The pH value of the buffer solution used for protein immobilization is of great importance. The pH value should be optimal for the reaction between the activated carboxyl groups of MUA and amino groups on the surface of the immobilized protein. In addition, the pre-concentration stage, which consists of increasing the local concentration of protein near the surface of the SPR sensor, contributes to the efficiency of immobilization. If the sensor surface and the protein have opposite charges, resulting in electrostatic interaction between them, the local concentration of protein can be further increased. A pH value of 0.5–1 less than the isoelectric point (pI) is required to obtain a positive charge on the amino groups of the immobilized protein, which implies that proteins with higher pI values are better suited for this type of immobilization [35]. The pKa value of MUA is lower than 4.4 [36], and therefore a buffer pH value higher than 4.4 was chosen to obtain a negative charge on the carboxyl groups of MUA. Experiments were performed in the pH range from 4.5 to 5.8, in which the SCoV2-rN protein (pI = 10.07 [37]) has a positive charge at which electrostatic interaction with the sensor surface occurs. The optimal pH value was determined by monitoring the SPR angle shift after the interaction of the immobilized SCoV2-rN protein with anti-SCoV2-rN (Figure 3).

As can be seen, the pH value had a high impact on the SPR signal, which was registered as a response to the interaction of the immobilized SCoV2-rN protein with specific antibodies present in the solution. The largest SPR angle shift (90.1 ± 4.5 m°) was registered using acetate buffer, pH 5.3, and was significantly different from values registered using buffers for which the pH values were equal to 5.0 (76.5 ± 9.0 m°) and 5.8 (75.4 ± 3.7 m°), respectively. For further experiments, 50 mM acetate buffer, pH 5.3, was used.

**Figure 4:**
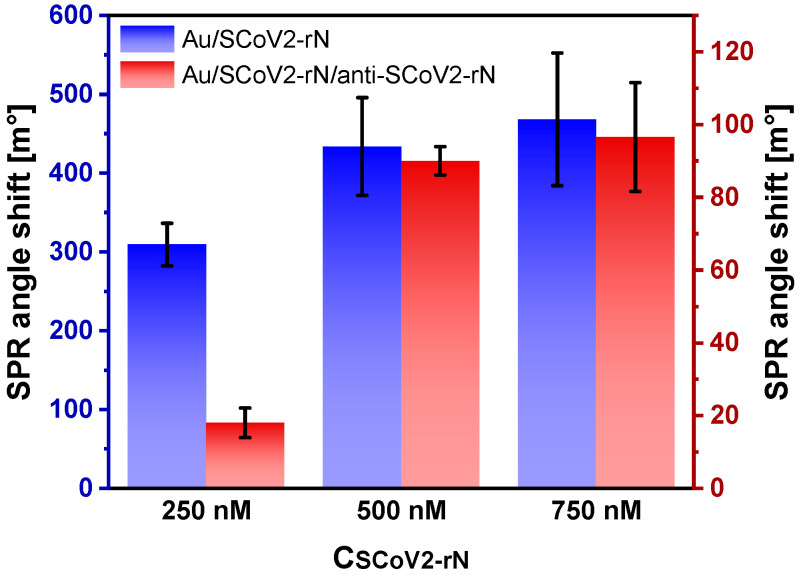
Effect of initial SCoV2-rN concentration on the magnitude of the response to SCoV2-rN immobilization and interaction with anti-SCoV2-rN. Conditions: SCoV2-rN was diluted in 50 mM acetate buffer, pH 5.3; 15 nM anti-SCoV2-rN. Error bars represent the standard deviation of three measurements (n = 3).

The optimal surface density of immobilized protein is critical to obtain the highest SPR analytical signal. Protein aggregation on the surface of the SPR sensor and steric hindrance to antibody binding can occur if excessive amounts of protein are immobilized. On the other hand, a low surface coverage may be the cause for the low SPR signal detected upon antibody binding [38]. Thus, a larger surface density does not always mean a larger SPR signal. The pH value of the buffer used for immobilization and the initial concentration of protein are equally important for achieving optimal density. The optimal concentration of SCoV2-rN was evaluated by SPR angle shifts registered after immobilization and after interaction with anti-SCoV2-rN (Figure 4). Increasing the initial SCoV2-rN concentration from 250 to 500 nM resulted in an increase in the SPR signal recorded after immobilization from 309 ± 27 to 434 ± 62 m°, with a much higher increase in the amount of bound anti-SCoV2-rN, as evidenced by a five-fold increase in the recorded signal to 90 ± 3.9 m°. In the case of an even higher initial concentration of SCoV2-rN (750 nM), the registered SPR angle shifts were slightly higher but were not statistically significantly different. Thus, the optimal SCoV2-rN concentration can be considered to be 500 nM, at which the SCoV2-rN protein mass concentration at the surface was 3.61 ± 0.52 ng/mm^2^, considering that a 1 ng/mm^2^ change in surface mass concentration results in a measured 120 m° change in SPR angle.

**Figure 5:**
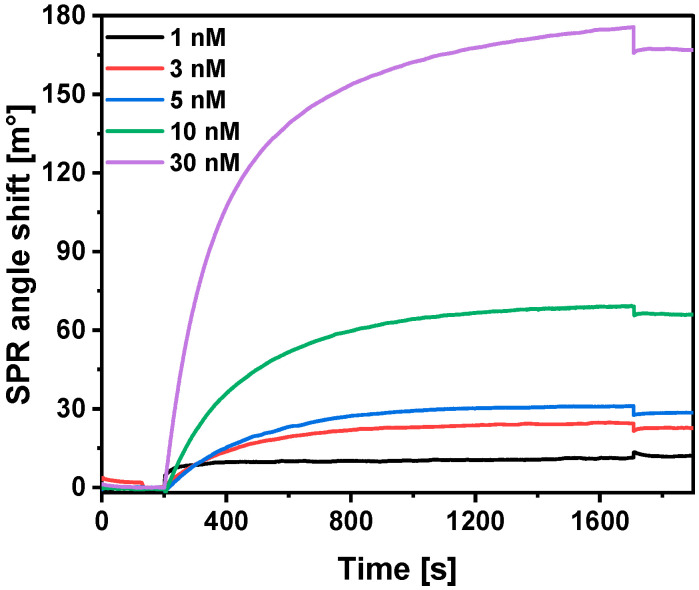
SPR kinetic study of immune complex formation between immobilized SCoV2-rN protein and anti-SCoV2-rN present in the buffer. Conditions: 500 nM SCoV2-rN; interaction time—1500 s; 1, 3, 5, 10, and 30 nM anti-SCoV2-rN.

Since mouse monoclonal antibodies were chosen as the analyte, it was decided to investigate their affinity to the immobilized SCoV2-rN protein. The affinity of antibody-antigen binding can be evaluated from the SPR kinetic study [39] by calculation of *K_D_*. High affinity is evidenced by the low value of *K_D_*. The interaction of anti-SCoV2-rN in the concentration range from 1 to 30 nM with immobilized SCoV2-rN protein was investigated. Equilibrium analysis requires reaching the necessary equilibrium under steady-state conditions [38]; therefore, the interaction was extended up to 1500 s (Figure 5). *K_D_* and *B_max_* were calculated to be 6.49 × 10^−8^ ± 5.3 × 10^−9^ M and 559 ± 33 m°, respectively. Such values indicate a sufficiently strong affinity between the selected antigen–antibody pair.

One of the advantages of SPR spectroscopy is the ability to quantify an analyte in solution without a label. This direct immunoassay format is a simple detection method that provides reliable real-time measurement results. In addition, this detection option is not costly or time-consuming [40].

**Figure 6:**
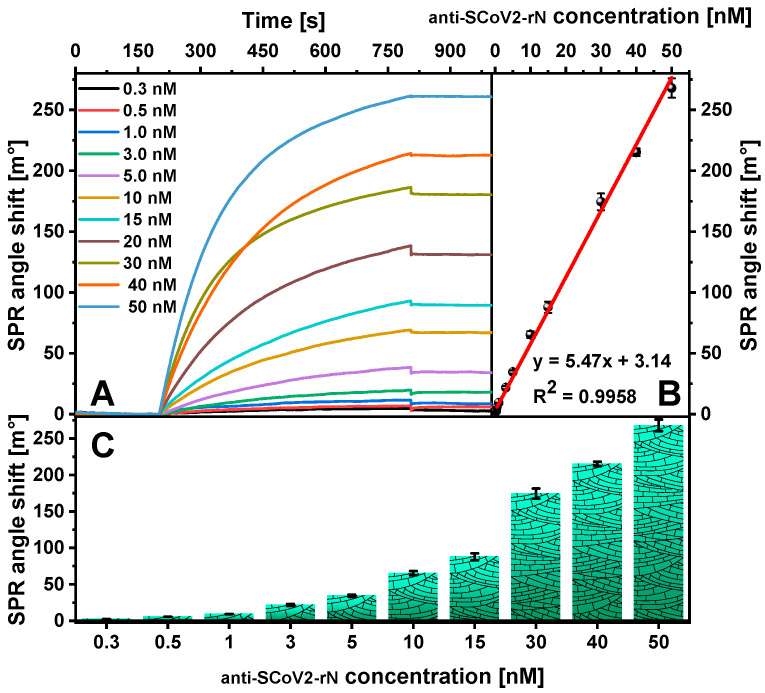
(**A**) SPR sensograms recorded during the analysis of solutions with different anti-SCoV2-rN concentrations using a direct immunoassay format and (**B**) a calibration curve. (**C**) Dependence of SPR angle shift on anti-SCoV2-rN concentration. Conditions: 500 nM SCoV2-rN; interaction time—600 s; 0.3, 0.5, 1, 3, 5, 10, 15, 20, 30, 40, and 50 nM of anti-SCoV2-rN. Error bars represent the standard deviation of three measurements (n = 3).

The ability to detect anti-SCoV2-rN using the developed SPR immunosensor was tested over a concentration range of 0.3 to 50 nM. The association phase was carried out for 600 s. The sensograms obtained during the monitoring of the affinity binding of anti-SCoV2-rN are shown in Figure 6A. The SPR response was calculated and plotted against anti-SCoV2-rN concentration (Figure 6B). The increase in SPR angle with increasing anti-SCoV2-rN concentration was obtained. A linear relationship (R^2^ = 0.9958) (Figure 6C) between the SPR angle shift and the concentration of anti-SCoV2-rN was found to be between 0.5 and 50 nM (75 to 7500 ng/mL). Decreasing the antibody concentration to 0.3 nM resulted in deviations from linearity. The limit of detection (LOD) and limit of quantification (LOQ) were calculated as 0.057 and 0.19 nM (8.55 and 28.5 ng/mL) at a signal-to-noise ratio of 3 and 10, respectively.

Analytical characteristics of the developed immunosensor were compared with other immunosensors designed for the detection of antibodies against the N protein (Table 1). This comparison is not entirely valid because different antibodies, whose affinity and activity vary, were used as analytes. For example, in the case of polyclonal antibodies, more antibodies should bind to the different epitopes of antigen, resulting in higher sensitivity. Immunosensors with electrochemical transducers [14,20] were characterized by lower LOD values than others. The LOD values in other studies were similar or higher than the calculated LOD in this work. An SPR-based immunosensor [25] had a more than five-fold higher LOD, although the detection of a polyclonal antibody should allow one to hope for higher sensitivity. Comparison with other studies [26,27] that also used SPR spectroscopy for the detection of specific antibodies is not possible because they did not report analytical parameters in comparable units (in serum dilutions). In addition, the concentration of polyclonal antibodies against the N protein in patient serum has been reported to be in the μg/mL range [41], so the linear range obtained in this study should be sufficient for the determination of specific antibodies in real samples.

**Figure 7:**
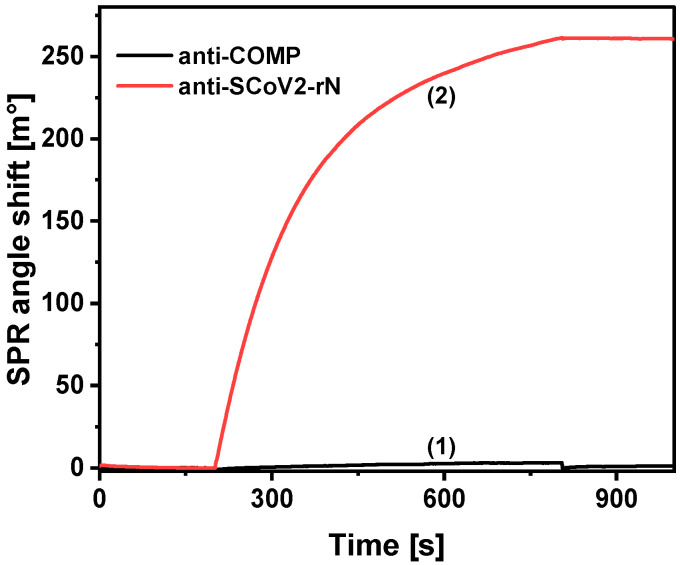
Non-specific interaction study. The SPR signal registered during (**1**) non-specific binding of anti-COMP antibodies on the SCoV2-rN-modified SPR sensor chip surface, and (**2**) formation of the immune complex of immobilized SCoV2-rN and anti-SCoV2-rN antibodies present in the sample. The study was performed by injecting 50 nM of anti-COMP or anti-SCoV2-rN in 10 mM PBS solution, pH 7.4.

The repeatability of the fabricated immunosensor was evaluated. The relative standard deviation (RSD) was 4.5% when the solution containing 7 nM anti-SCoV2-rN was measured five times using the same SPR sensor chip. RSD values ranged from 1.2 to 5% when different concentrations of anti-SCoV2-rN were measured using several equally modified SPR sensor chips. The coefficients of variation of the intra-assay and inter-assay were equal to 2.8 and 3.5%, respectively. This indicates good repeatability of the immunosensor. Finally, the specificity of the fabricated immunosensor was evaluated. For this purpose, non-specific interaction of antibodies against cartilage oligomeric matrix protein (50 nM anti-COMP; the same isotype as anti-CoV2-rN antibodies) on the Au/SCoV2-rN surface was tested (Figure 7). Injection of 50 nM anti-COMP solution onto the surface resulted in 2.8 ± 1.3 m° of the measured analytical signal. Considering the 267.95 ± 7.99 m° of signal registered using 50 nM anti-SCoV2-rN, it can be noted that the non-specific binding is very low. Moreover, further experiments were performed with 0.1 M HEPES (2-[4-(2-hydroxyethyl)piperazin-1-yl]ethane-1-sulfonic acid) buffer as a one of biological buffers. No effect of HEPES as an organic compound with zwitterionic properties on the registered signal was observed. It can be summarized that the fabricated immunosensor has good specificity.

## 3. Materials and Methods

### 3.1. Reagents

SCoV2-rN (SARS-CoV-2 recombinant nucleocapsid protein expressed in yeast *Saccharomyces cerevisiae*, C-terminal His6-tag) and anti-SCoV2-rN antibodies (mouse monoclonal antibodies (cl. B12N), protein A purified) were purchased from Baltymas (Vilnius, Lithuania). Antibodies against cartilage oligomeric matrix protein (monoclonal mouse IgG1 clone 16F12, purified by affinity chromatography) were acquired from BioVendor (Brno, Czech Republic). Acetic acid (CH_3_COOH), hydrochloric acid 37% (HCl), sulfuric acid 98% (H_2_SO_4_), phosphate buffered saline (PBS), pH 7.4, tablets, glycine (C_2_H_5_NO_2_), magnesium chloride hexahydrate (MgCl_2_ · 6H_2_O), sodium hydroxide (NaOH), sodium dodecyl sulfate (SDS), sodium acetate (CH_3_COONa), hydrogen peroxide (H_2_O_2_), N-(3-dimethylaminopropyl)-N’-ethylcarbodiimide hydrochloride (EDC), ethanolamine (C_2_H_7_NO), and 2-[4-(2-hydroxyethyl)piperazin-1-yl]ethane-1-sulfonic acid (HEPES) were purchased from Carl Roth (Karlsruhe, Germany). Ethanol 99.9% (C_2_H_5_OH) was obtained from Honeywell (Hesse, Germany). N-hydroxysuccinamide (NHS) was purchased from Alfa Aesar (Karlsruhe, Germany). The 11-mercaptoundecanoic acid (MUA) was obtained from Sigma-Aldrich (Darmstadt, Germany). The argon gas (Ar) used to dry the gold-coated SPR sensor chip was purchased from Esme Messer Gaas (Vilnius, Lithuania).

### 3.2. Gold-Coated Sensor Chip Preparation

Adequate preparation of the gold-coated SPR sensor chip is necessary before measurements can be taken. The sensor chip was cleaned by immersion in a Piranha solution prepared by mixing solutions of H_2_O_2_ and H_2_SO_4_ in the ratio of 1:3. The sensor chip was kept in the Piranha solution for 5 min at a controlled temperature of 60 °C. After that, the sensor chip was washed carefully with distilled water, incubated in ethanol for 2 min, then washed again with distilled water and dried with argon gas. A SAM was then formed on the surface of the sensor chip using a previously applied methodology [36,42]. For this purpose, the dried sensor chip was incubated for 24 h in 4 mL of 1 mM MUA ethanol-based solution. To ensure the formation of a monolayer rather than aggregates [43], the chip was removed from the MUA solution, rinsed with distilled water, left in ethanol for 2 min, rinsed again with distilled water, and dried with argon gas. The sensor chip modified by MUA SAM (Au/MUA) was deposited on a hemicylinder coated with a refractive index matching fluid. The hemicylinder assembled on the slider was inserted into the SPR instrument (Autolab ESPRIT, Utrecht, The Netherlands) having two separate channels with a surface area of 7.9 mm^2^.

### 3.3. Gold-Coated Sensor Chip Surface Stabilization

Before covalent SCoV2-rN immobilization, the Au/MUA surface had to be stabilized/rehydrated. For this purpose, the sensor chip surface was treated with two solutions: a regeneration solution consisting of 10 mM NaOH with 0.5% SDS and 50 mM acetate buffer solution, pH 5.3. A computer program controlled the SPR instrument by alternately injecting the respective solutions into a cuvette on the surface of the sensor chip with an interval of 120 s between injections. The stabilization process was continued until the baseline stabilized (at least 30 min).

### 3.4. Nucleocapsid Protein Immobilization

Covalent immobilization of SCoV2-rN was performed identically in both channels of the cuvette. The first step was stabilization with 50 mM acetate buffer, pH 5.3, for 200 s. In the second step, the carboxyl groups of MUA were activated with a solution containing 0.2 M EDC and 0.05 M NHS for 600 s. This resulted in the formation of the active O-acylisourea intermediate, which could then be readily displaced by nucleophilic attack of the primary amino groups in the immobilized protein. In the third step, a brief washout procedure was conducted to remove excess EDC/NHS from the surface. In the fourth step, covalent immobilization of SCoV2-rN was carried out for 1200 s. SCoV2-rN solution of a certain concentration in 50 mM acetate buffer, pH 5.3, was injected into both channels of the cuvette. In the fifth step, unbound protein molecules were washed from the sensor chip surface. In the sixth step, deactivation was performed for 600 s. A 1 M ethanolamine solution, pH 8.5, was injected into both channels. This solution deactivated the remaining active carboxyl groups. The ethanolamine solution was washed out, and a regeneration solution of 10 mM NaOH with 0.5% SDS was added, followed by 50 mM acetate buffer, pH 5.3. Finally, the stabilization procedure was performed to obtain SCoV2-rN immobilized on the Au/MUA surface (Au/SCoV2-rN).

### 3.5. Interaction of Immobilized SCoV2-rN with Specific Antibodies

To achieve interaction of the SCoV2-rN with anti-SCoV2-rN, the process started with stabilization using 10 mM PBS solution, pH 7.4, in both channels of the cuvette for 200 s. The first channel was then filled with an antibody solution of the appropriate concentration in 10 mM PBS solution, pH 7.4, and 10 mM PBS solution, pH 7.4, was injected into the second channel, which was used as a reference channel. The interaction was carried out for 600 s resulting in the formation of an immune complex (Au/SCoV2-rN/anti-SCoV2-rN). This was followed by a dissociation phase performed by injection of 10 mM PBS solution, pH 7.4, and further stirring for 200 s, during which unbound antibodies were washed out of the cuvette. Finally, during 500 s, a regeneration process occurred in which the interaction between the covalently bound protein and the antibodies was broken, and further baseline was registered in 10 mM PBS solution, pH 7.4.

### 3.6. Selection of Regeneration Solution and Regeneration Duration

Several regeneration solutions have been thoroughly tested and evaluated to determine the greatest effectiveness. Solutions containing (1) 10 mM NaOH and 0.5% SDS; (2) 25 mM NaOH and 0.5% SDS; (3) 50 mM NaOH and 0.5% SDS; (4) 10 mM glycine, pH 2.0; (5) 10 mM glycine, pH 3.0; (6) 10 mM glycine, pH 4.0; or (7) 1 M MgCl_2_ were tested as a potential regeneration solution. The solutions tested varied in chemical composition, pH value, and ionic strength, each of which may affect the regeneration process differently. By studying the regeneration efficiency, it is possible to determine which regeneration solution produces the most desirable results, providing high-quality data for subsequent stages of the experiment.

After determining a suitable regeneration solution for the experiments, the next responsible step was to carefully select the optimal regeneration duration. For this purpose, regeneration using 10 mM NaOH and 0.5% SDS was carried out for 100, 300, 500, and 600 s.

### 3.7. Optimization of SCoV2-rN Surface Concentration

The SCoV2-rN concentration optimization process began with the immobilization of the SCoV2-rN protein, as described previously. After immobilization, the interaction of SCoV2-rN and specific antibodies was carefully studied. To determine the optimal concentration, a series of experiments were performed with three different SCoV2-rN concentrations: 250, 500, and 750 nM. The SCoV2-rN protein was diluted in 50 mM acetate buffer, pH 5.3.

### 3.8. Calculations

The SPR response was calculated as the SPR angle shift recorded before and after immobilization of the SCoV2-rN protein or its interaction with anti-S-CoV2-rN. The difference between the SPR angle value in the first and second channel was used as SPR angle shift, then the second channel was used as a reference, with a 120 m° change in SPR angle corresponding to a change in mass concentration at the surface of approximately 1 ng/mm^2^.

The regeneration efficiency was calculated by dividing the SPR signal recorded at baseline before interaction between S-CoV2-rN and anti-S-CoV2-rN and the SPR signal after treatment with the regeneration solution. The equilibrium dissociation constant (*K_D_*) and surface-binding capacity (*B_max_*) were calculated according to a single site interaction model [44]: *B_eq_* = *B_max_* × ([*Ab*]/([*Ab*] + *K_D_*), where *B_eq_* is the equilibrium SPR response under steady-state conditions, and [*Ab*] is the concentration of anti-S-CoV2-rN.

To calculate the limit of detection (LOD), the concentration of anti-S-CoV2-rN at which the SPR signal is equal to three standard deviations from the baseline noise was estimated. The LOD is a critical parameter because it indicates the lowest antibody concentration that can be reliably detected and distinguished from background noise in the SPR measurements. In addition, the limit of quantification (LOQ) is evaluated by determining the concentration of anti-S-CoV2-rN that yields the SPR signal equivalent to 10 standard deviations of the baseline noise. The LOQ represents the minimum concentration at which the SPR signal can be accurately quantified, providing a threshold for reliable quantification of the antibody.

One-way analysis of variance (ANOVA) at a 95% confidence level was used to analyze the significance of differences between some obtained results. Intra-assay coefficient of variation (CV) was calculated for measurements performed using a single modified SPR chip (two measurements for six individual samples). Results obtained using several equally modified SPR chips on different days were used for inter-assay CV calculations (two measurements for 10 individual samples).

## 4. Conclusions

In this work, we proposed to use SPR spectroscopy for the direct, real-time detection of anti-SCoV2-rN antibodies which does not require the use of additional labels and thus makes quantification simpler and shorter. The optimal conditions for the fabrication of the immunosensor were achieved using 500 nM of SCoV2-rN in 50 mM acetate buffer solution, pH 5.3, providing the surface mass concentration of SCoV2-rN of 3.61 ± 0.52 ng/mm^2^, which improves the analytical performance of the immunosensor. Regeneration using 10 mM NaOH and 0.5% SDS for 500 s was found to be optimal for dissociation of the formed SCoV2-rN/anti-SCoV2-rN immune complex. Optimization of the immunosensor fabrication allowed us to achieve LOD and LOQ values of 0.057 and 0.19 nM, respectively, with a linear range from 0.5 to 50 nM. In addition, the developed immunosensor is suitable for multiple anti-SCoV2-rN antibody detections.

Although the analytical characteristics of the fabricated immunosensor were tested with mouse monoclonal antibodies, the proposed SPR immunosensor can be successfully applied for the detection of human antibodies against the SARS-CoV-2 nucleocapsid protein without the need for the labelled molecules.

## Figures and Tables

**Table 1:** Comparison of the analytical performance of some immunosensors designed for the detection of antibodies against SARS-CoV-2 nucleocapsid protein.

Antibody Type	Detection Method	Linear Range [ng/mL]	LOD [ng/mL]	Reference
n/a	electrochemical impedance transduction	−	0.00195 (13 fM)	[14]
Monoclonal humanized IgG	paper-based ELISA	1–50	9	[18]
Rabbit polyclonal IgG	electrochemical immunosensor	1 × 10^−6^–1	1 × 10^−6^	[20]
n/a	lateral flow immunoassay	−	5	[22]
Polyclonal IgG	SPR spectroscopy	100–10,000	45.6	[25]
Mouse monoclonal IgG	SPR spectroscopy	75–7500	8.55	This work

## Data Availability

The data presented in this study are available on request.
